# Supplementary material for: Genetic identification of the functional surface for RNA binding by Escherichia coli ProQ
Source: Nucleic Acids Res. 2020 Mar 14;48(8):4507–20. doi: 10.1093/nar/gkaa144 (PMC7192607; doi:10.1093/nar/gkaa144)
Supplement: gkaa144_Supplemental_Files [file gkaa144_supplemental_files.zip › Pandey_et_al_revised_Supplement.pdf]

Supplementary Materials for

Genetic identification of the functional surface for RNA binding by *Escherichia coli* ProQ

Smriti Pandey, Chandra M. Gravel, Oliver M. Stockert, Clara D. Wang, Courtney L. Hegner, Hannah LeBlanc, and Katherine E. Berry

This pdf file includes:

Supplementary Figures S1 to S12

Supplementary Tables S1 to S6

Supplementary References

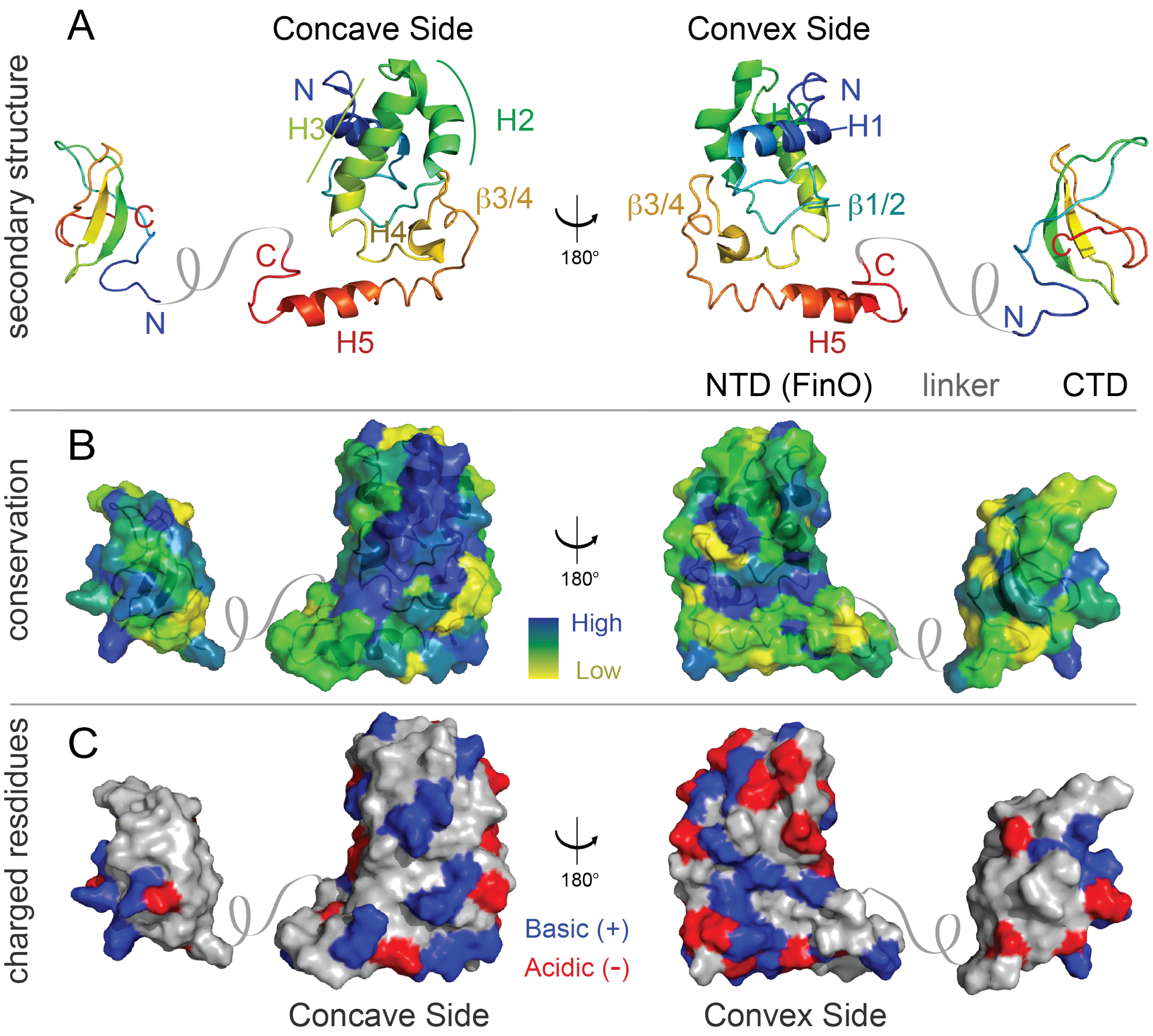

Supplementary Figure 1. Structure of *E. coli* ProQ NTD and CTD. (A) Cartoon representation of ProQ NTD (resi: 1-119, PDB ID: 5nb9) and CTD (resi: 180-232, PDB ID 5nbb) NMR structures (colored as rainbow chains (N-terminus: blue → C-terminus: red).(9) The less conserved linker domain (resi 120-179) is depicted as a hand-drawn grey coil. The NTD and CTD are shown in an arbitrary arrangement to one another. The right-hand side shows the same structures viewed from a 180° angle. N and C termini are indicated, as are the positions of key secondary-structure elements, as previously defined: α helices (H)1-5,(10) and β hairpins β1/2 and β3/4.(3) (B) Surface representations of ProQ NTD and CTD, colored according to degree of conservation (highly conserved: blue → minimally conserved: yellow). Conservation was determined by ConSurf server (8) using PDB ID 5nb9 as an input. (C) Surface representations of ProQ NTD and CTD, colored by electrostatic nature of side chains. Acidic residues (Asp and Glu), predicted to be negatively charged at cellular pH, are colored red; basic residues (Lys and Arg), predicted to be positively charged at cellular pH, are colored blue. Remaining surface residues are colored grey.

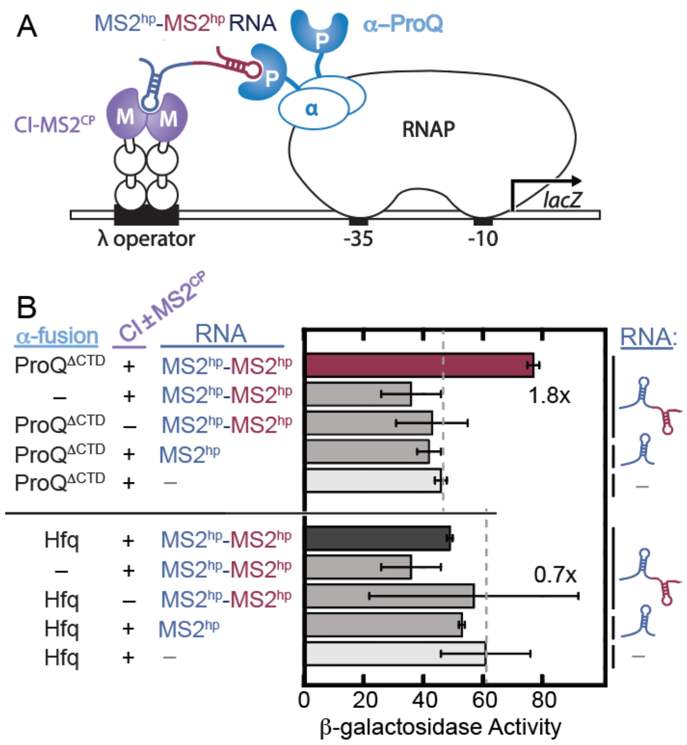

Supplementary Figure 2. Interaction of ProQ with an MS2^{hp} in B3H assay. (A) Schematic depicting how an RNA with two MS2^{hp} moieties could bridge an interaction between the CI-MS2^{CP} and α-ProQ if ProQ interacts with one of the two hairpins. B) Results of a representative β-galactosidase assay performed with reporter-strain cells containing three compatible plasmids: one (α-fusion) that encoded α alone (–) or an α-fusion protein (α-ProQ$^{\Delta CTD}$ or α-Hfq), another (CI±MS2^{CP}) that encoded λCI alone (–) or the λCI-MS2^{CP} fusion protein (+), and a third (RNA) that encoded an RNA containing 2, 1, or 0 copies of the MS2^{hp} moiety (MS2^{hp}-MS2^{hp}, MS2^{hp} or –, respectively). Cells were grown in the presence of 0.2% arabinose and 50 μM IPTG. The bar graphs show absolute β-gal values as the averages and standard deviations of biological triplicate measurements. Each interaction is labeled with the fold-stimulation over basal levels (see Methods).

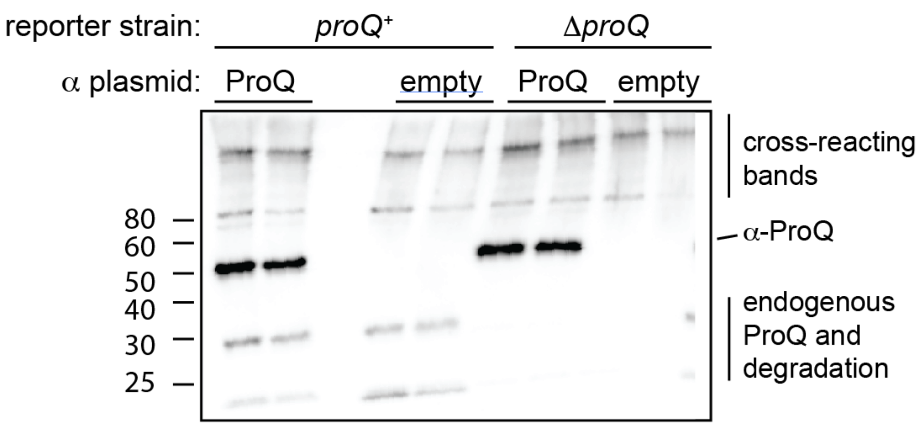

Supplementary Figure 3. ProQ Western Blot and Identification of bands from B3H lysates. Lysates were taken from β-galactosidase assays performed with *proQ*$^{+}$ (KB480) or Δ*proQ (*SP2) reporter strain cells containing three compatible plasmids: one the CI-MS2^{CP} fusion protein, another that encoded α (empty) or the α-ProQ$^{\Delta CTD}$ fusion protein (ProQ), and a third that encoded an MS2^{hp}-*cspE* hybrid RNA. Cells were grown in the presence of 0.2% arabinose and 10 μM IPTG, lysed, electrophoresed and blotted with an anti-ProQ polyclonal antibody (see Methods). The positions of molecular weight markers are indicated on the left. Assignments of bands to particular ProQ species are indicated on the right. Endogenous ProQ is expected to run at 26 kDa and the α-ProQ$^{\Delta CTD}$ fusion protein at 48 kDa. The identities of these bands are further confirmed by their disappearance from lysates from a Δ*proQ* reporter (missing endogenous ProQ) or with an α-empty plasmid (missing α-ProQ$^{\Delta CTD}$). The fact that the signal from α-ProQ is much higher than that arising from either cross-reacting bands or endogenous ProQ allowed for a dot-blot assay to meaningfully report on α-ProQ levels without prior electrophoresis (see Fig S7).

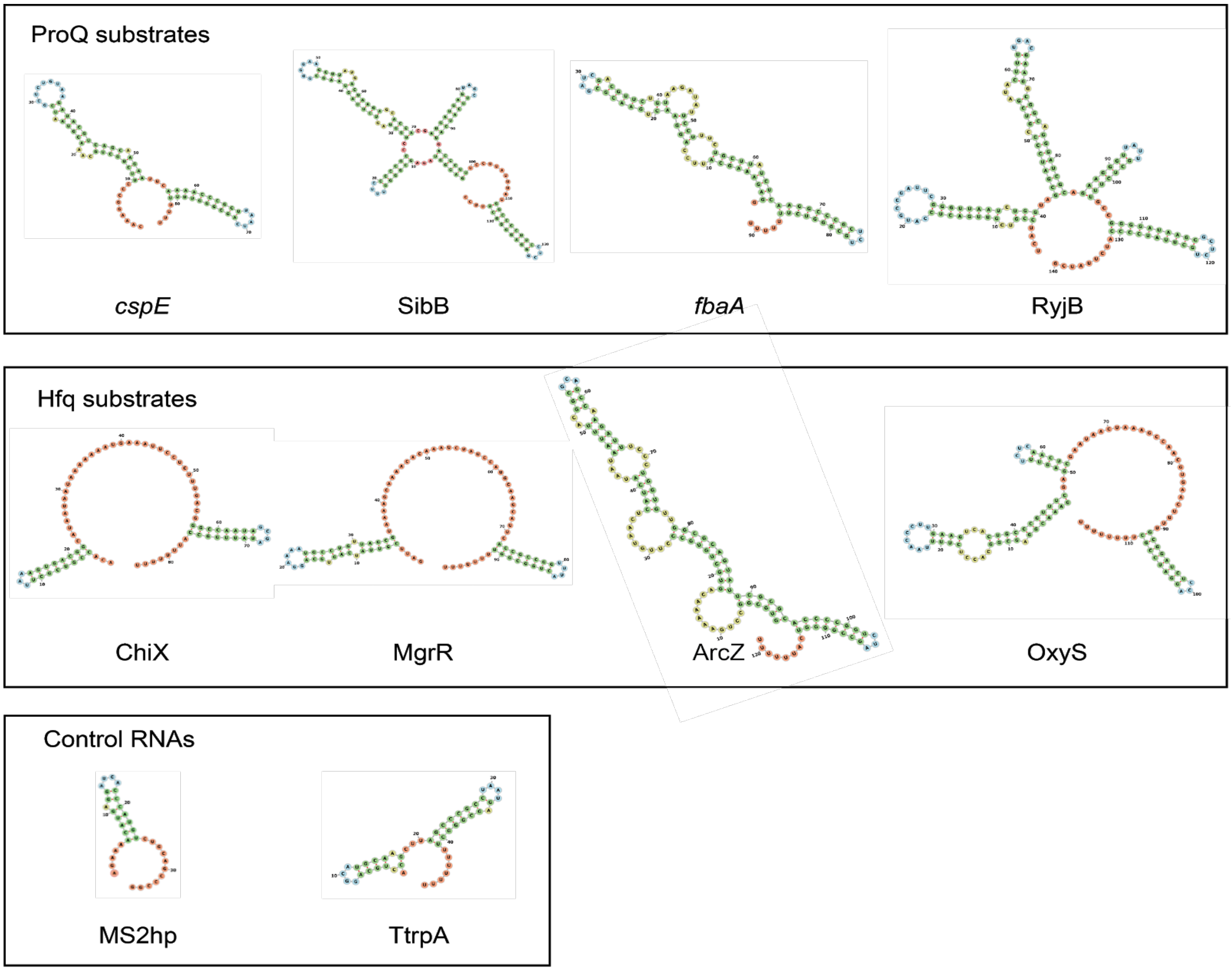

Supplementary Figure 4. Predicted secondary structures of RNAs tested in B3H assay. The predicted secondary structures of RNA sequences cloned into 1XMS2^{hp}-hybrid-RNA constructs and tested for B3H interactions in Fig 2. RNAs are separated according to whether they were selected for study based on literature evidence that they interact with ProQ (ProQ substrates), are Hfq-dependent sRNAs (Hfq substrates) or controls RNAs (see Results). Secondary structures were predicted with an RNA Fold algorithm and displayed using Forna.(6, 7) Complete sequences of each hybrid RNA are provided in Table S4.

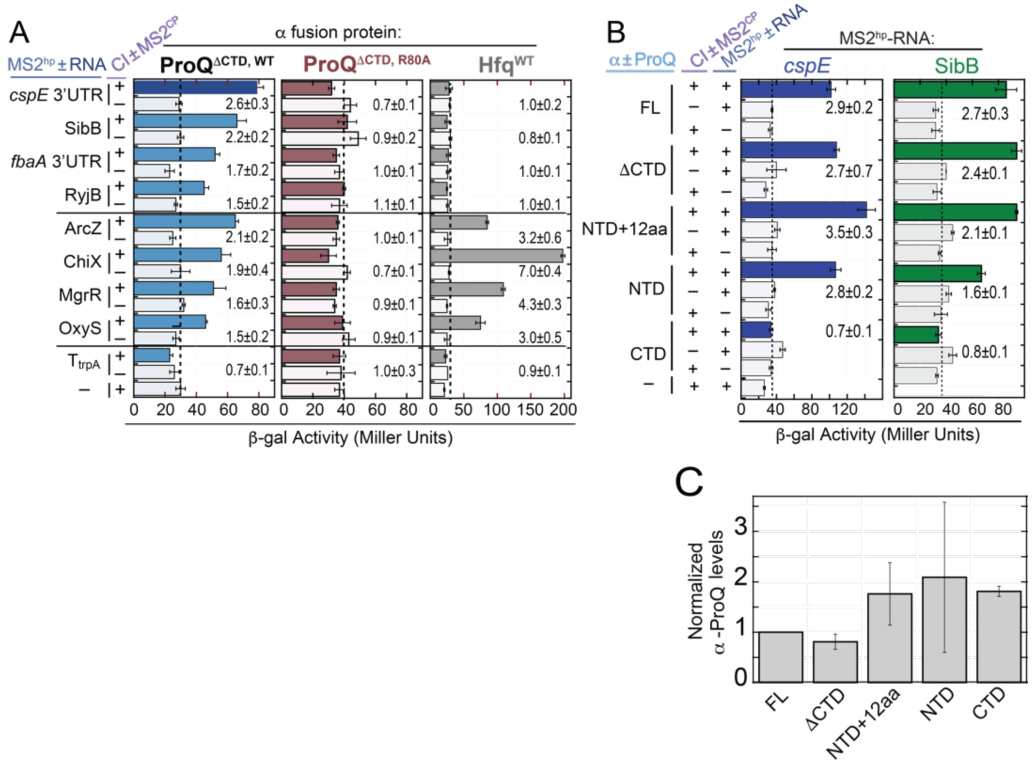

Supplementary Figure 5. Absolute β-galactosidase values from representative datasets. β-galactosidase (β-gal) values from which fold-stimulation over basal levels were calculated for data presented (A) Figure 2 and (B) Figure 3. (A) Results of a representative B3H assay between a panel of RNA substrates with wild type α-ProQ$^{WT\Delta CTD}$, α-ProQ$^{R80A\Delta CTD}$ or α-Hfq. β-gal assays were performed with *Δhfq* reporter strain cells containing three compatible plasmids: one (CI±MS2^{CP}) that encoded λCI (-) or the λCI-MS2^{CP} (+) fusion protein, another that encoded an α-fusion protein (α-ProQ$^{\Delta CTD}$, either with wild type ProQ or an R80A mutant, or α-Hfq), and a third (MS2^{hp}±RNA) that encoded a hybrid RNA (a single MS2^{hp} moiety fused to *cspE* 3' UTR, SibB, *fbaA* 3' UTR, RyjB, ArcZ, ChiX, MgrR, OxyS, trpA terminator (T_{trpA}) or an RNA that contained only the MS2^{hp} moiety (-). (B) Results of a representative B3H assay detecting interactions between α-ProQ truncations and *cspE* and SibB RNAs. β-gal assays were performed with *Δhfq* reporter strain cells containing three compatible plasmids: one (CI±MS2^{CP}) that encoded λCI (-) or the λCI-MS2^{CP} (+) fusion protein, another (α±ProQ) that encoded α alone (-) or an α-fusion protein (α-ProQ^{FL} (full-length; resi=2-232), α-ProQ$^{\Delta CTD}$ (resi=2-176), α-ProQ$^{NTD+12aa}$ (resi=2-131), α-ProQ^{NTD} (resi=2-119), or α-ProQ^{CTD} (resi=181-232)), and a third (MS2^{hp}±RNA) that encoded a hybrid RNA (MS2^{hp}-*cspE* or MS2^{hp}-SibB) or an RNA that contained only the MS2^{hp} moiety (-). For both panels, cells were grown in the presence of 0.2% arabinose and 50 µM IPTG. Bar graphs show absolute β-gal values as the averages and standard deviations of biological triplicate measurements. Each interaction is labeled with the fold-stimulation over basal levels (see Methods). (C) Quantification of Western Blots from Figure 3A. The intensity of each band (α-ProQ and endogenous RpoA) was quantified by densitometry. For each fusion construct, the intensity of α-ProQ was normalized to its corresponding endogenous-RpoA band. Each mutant was then normalized to full-length (FL) α-ProQ. The bar graphs show the normalized expression levels as averages and standard deviations of values collected from three experiments conducted across multiple days.

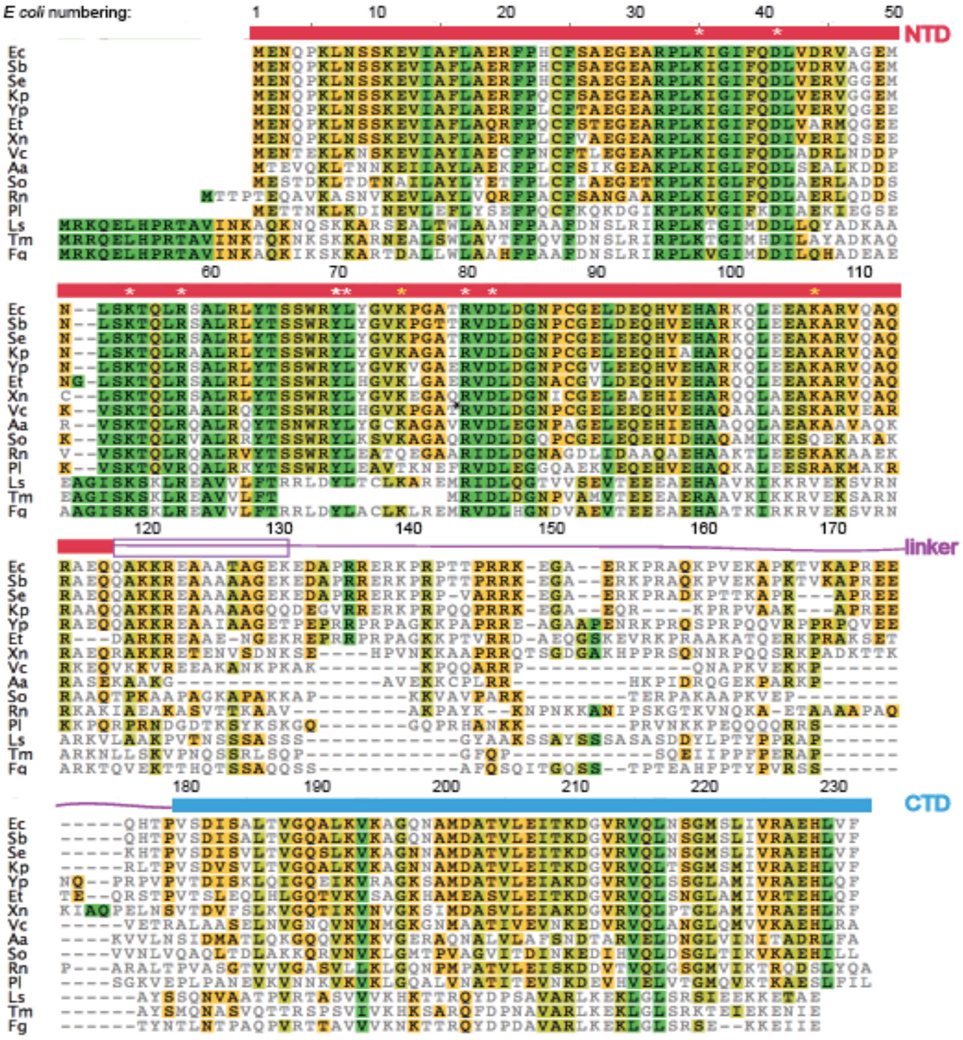

Supplementary Figure 6. Multiple sequence alignment of ProQ and FinO sequences. ProQ amino-acid sequences from the following species were obtained from NCBI: *Escherichia coli K12 (Ec), Shigella boydii (Sb), Salmonella enterica (Se), Klebsiella pneumoniae (Kp), Yersinia pestis (yp), Edwardsiella tardi (Et), Xenorhabdus nematophila (Xn), Vibrio cholerae (Vc), Aggregatibacter aphrophilus (Aa), Shewanella oneidensis (So), Rheinheimera nanhaiensis (Rn), Pseudoalteromonas luteoviolacea (Pl), Legionella shakespearei (Ls), Totlockia micdadei (Tm), Fluoribacter gormanii (Fg) Rheinheimera nanhaiensis*. Sequences were aligned using CLUSTALW with a BLOSUM cost matrix (reference). Residues are highlighted according to similarity (PAM120 score matrix; green, 100% similar; lime, 80-100% similar; yellow, 60% similar; no shading: 0-60% similar). Domain boundaries and residue numbers from *E. coli* ProQ are indicated above the sequences. Surface-exposed residues targeted for mutagenesis in this paper are indicated with an asterisk (white = highly conserved; yellow = less conserved). A purple box outlines the first 12 aa of the linker, which are included in the α-ProQ-NTD+12aa (resi=2-131) construct in Fig 3.

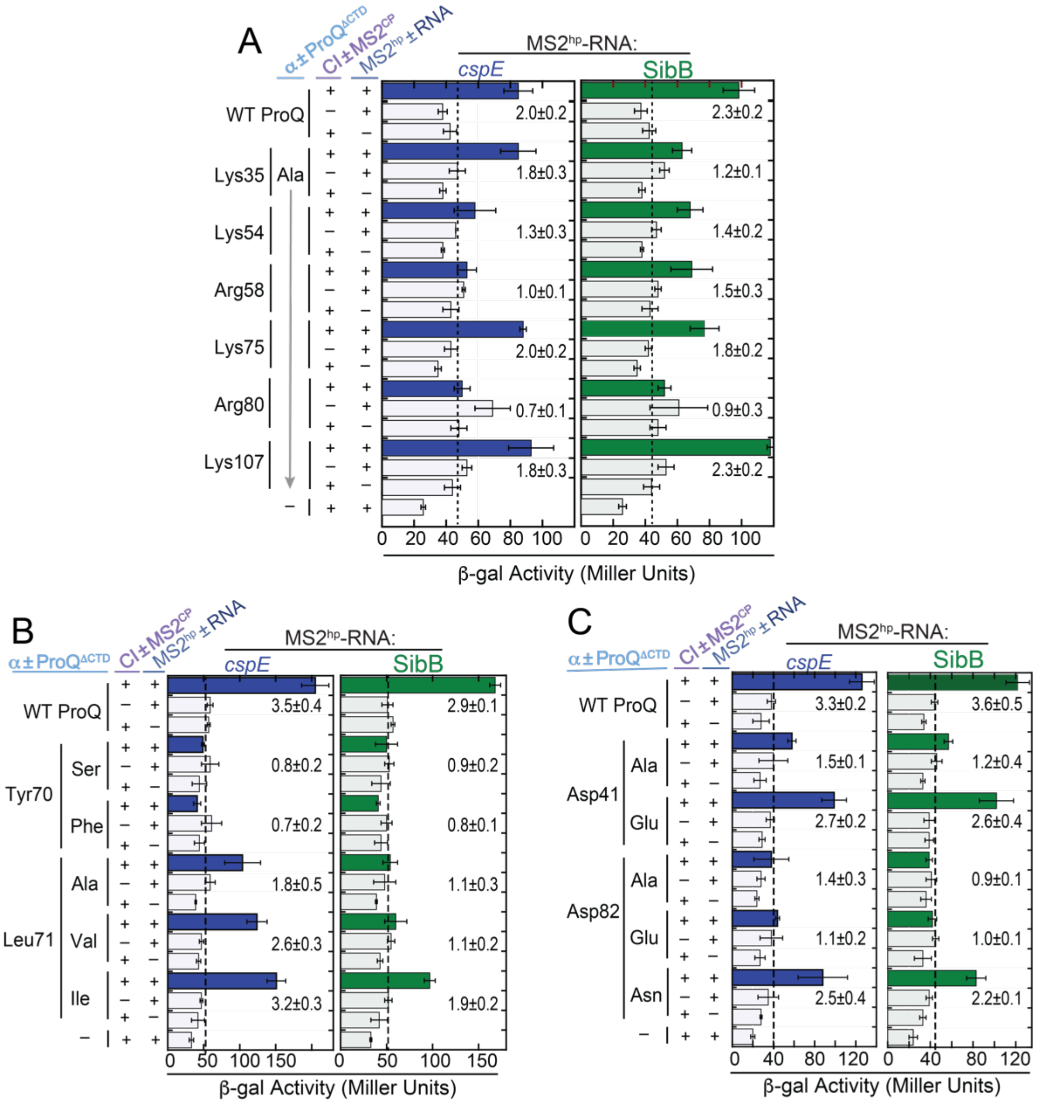

Supplementary Figure 7. Absolute β-galactosidase values of representative datasets. β-galactosidase (β-gal) values from which fold-stimulation over basal levels were calculated for data presented in Figure 4. Results of a representative B3H assays showing effects on ProQ-RNA interactions of alanine mutations at (A) basic, (B) hydrophobic and (C) acidic residues. β-gal assays were performed with *Δhfq* reporter strain cells containing three compatible plasmids: one (CI±MS2^{CP}) that encoded λCI (-) or the λCI-MS2^{CP} (+) fusion protein, another (α±ProQ) that encoded α alone (-) or an α-ProQ^{ΔCTD} fusion protein (wild type (WT) or the indicated mutant), and a third (MS2^{hp}±RNA) that encoded a hybrid RNA (1XMS2^{hp}-*cspE* or 1XMS2^{hp}-SibB) or an RNA that contained only the MS2^{hp} moiety (-). Cells were grown in the presence of 0.2% arabinose and 50 µM IPTG (A,B) or in the presence of 0.2% arabinose (C). We consistently see similar fold-stimulation values between the two conditions. The bar graphs show absolute β-gal values as the averages and standard deviations of biological triplicate measurements. Each interaction is labeled with the fold-stimulation over basal levels (see Methods).

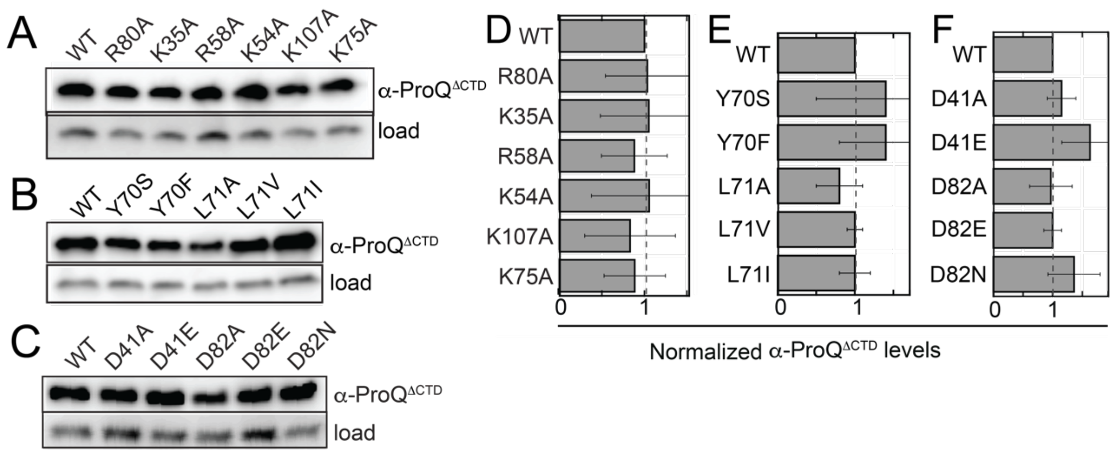

Supplementary Figure 8. Quantification of expression of α-ProQ$^{\Delta CTD}$ mutant fusion proteins. (A-C) Samples from Figure 4 D-F were analyzed by Western blot and probed with an anti-ProQ antibody detect α-ProQ$^{\Delta CTD}$ fusion protein. A cross-reacting band independent of the presence of endogenous ProQ or α-ProQ fusion protein is used as a loading control (load; see Fig S3). (D-F) The intensity of each band (α-ProQ$^{\Delta CTD}$ and load) was quantified by densitometry and the intensity of α-ProQ$^{\Delta CTD}$ was normalized to its corresponding load band. Values for each mutant were then normalized to wild type (WT) ProQ. The bar graphs show the normalized expression levels as averages and standard deviations of values collected from at least three experiments conducted across multiple days.

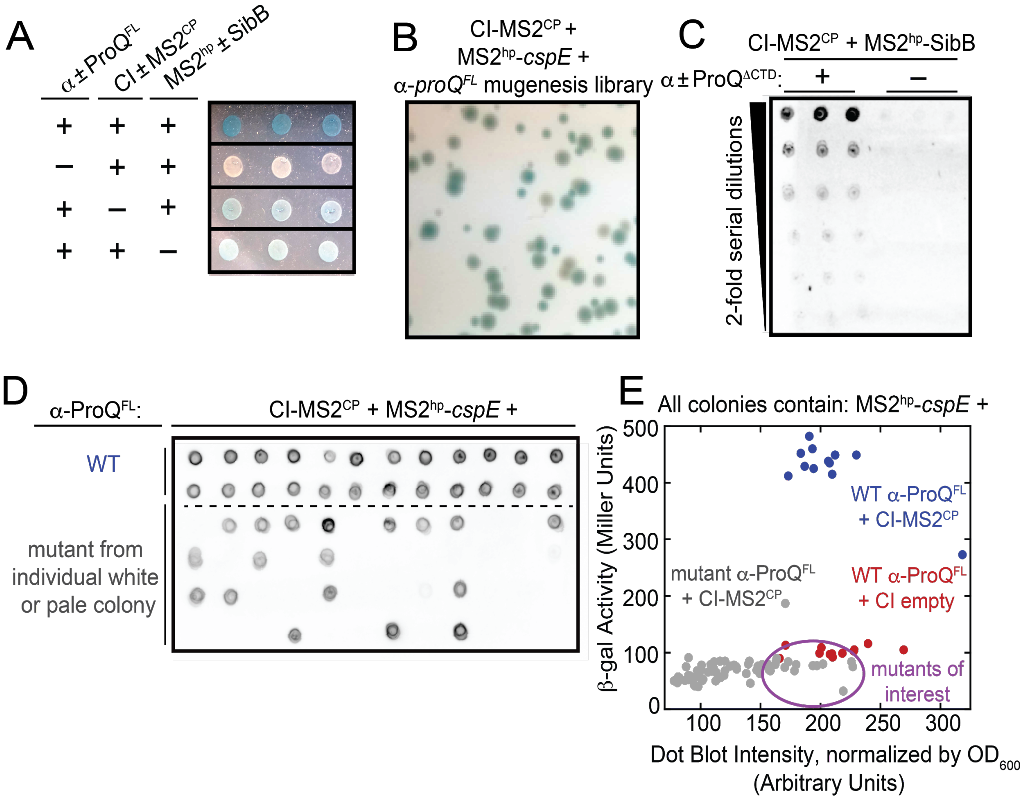

Supplementary Figure 9. Primary and Counter Screen Process. (A) Colony-color phenotypes of ProQ-SibB B3H interaction. Δ*hfq* reporter strain cells containing three compatible plasmids were spotted on solid medium: one (CI±MS2^{CP}) that encoded λCI (-) or the λCI-MS2^{CP} (+) fusion protein, another (α±ProQ^{FL}) that encoded α alone (-) or an α-ProQ^{FL} fusion protein (+), and a third (MS2^{hp}±SibB) that encoded a 1XMS2^{hp}-SibB hybrid RNA (+) or an RNA that contained only the MS2^{hp} moiety (-). Transformed cells were spotted on LB-agar medium containing 0.2% arabinose, 1.5 µM IPTG, 40 µg/mL X-gal and 125 µM TPEG and grown overnight at 37°C. Triplicate spots are shown. (B) Example of primary screen. Δ*hfq* reporter strain cells (SP5) were plated on LB-agar medium containing 0.2% arabinose, 1.5 µM IPTG, 40 µg/mL X-gal and 125 µM TPEG. Cells contained three compatible plasmids: one that encoded the CI-MS2^{CP} fusion protein, another that encoded an MS2^{hp}-*cspE* hybrid RNA, and a third that encoded a α-ProQ^{FL} mutagenesis library (see Methods). Plates were grown overnight at 37°C and then incubated at 4°C. (C) Establishment of sensitivity and background of anti-ProQ dot-blot assay. Lysates were taken from β-galactosidase (β-gal) assays performed with KB511 reporter-strain cells containing three compatible plasmids: one the λCI-MS2^{CP} fusion protein, another that encoded α (-) or the α-ProQ^{ΔCTD} fusion protein (+), and a third that encoded an MS2^{hp}-SibB hybrid RNA. Cells were grown in the presence of 0.2% arabinose and 25 µM IPTG; two-fold dilutions of lysate were spotted on a nitrocellulose membrane and blotted with an anti-ProQ polyclonal antibody (see Methods (D) Representative dot blot from counterscreen. White and pale colonies from primary screen (individual white or pale colonies) or control colonies containing plasmids encoding wild-type α-ProQ^{FL} along with CI-MS2^{CP} and MS2^{hp}-*cspE* (WT) were picked and grown in the presence of 0.2% arabinose and 0 µM IPTG (see Methods). Cells were lysed for β-gal analysis and lysate from each well was spotted on nitrocellulose and blotted with anti-ProQ antibody, as in (B). Densitometry measurements were conducted in ImageJ. (E) For each well, arising from a single colony, β-gal activity was plotted versus densitometry-determined dot-blot intensity, normalized by the optical density (OD_{600}) of the well when lysed (see Methods). Plasmids were miniprepped and sequenced from colonies, indicated with a purple oval, that contained a α-*proQ*^{FL} mutant sequence that gave rise to β-gal activity comparable to negative controls but anti-ProQ dot-blot intensity comparable to positive controls.

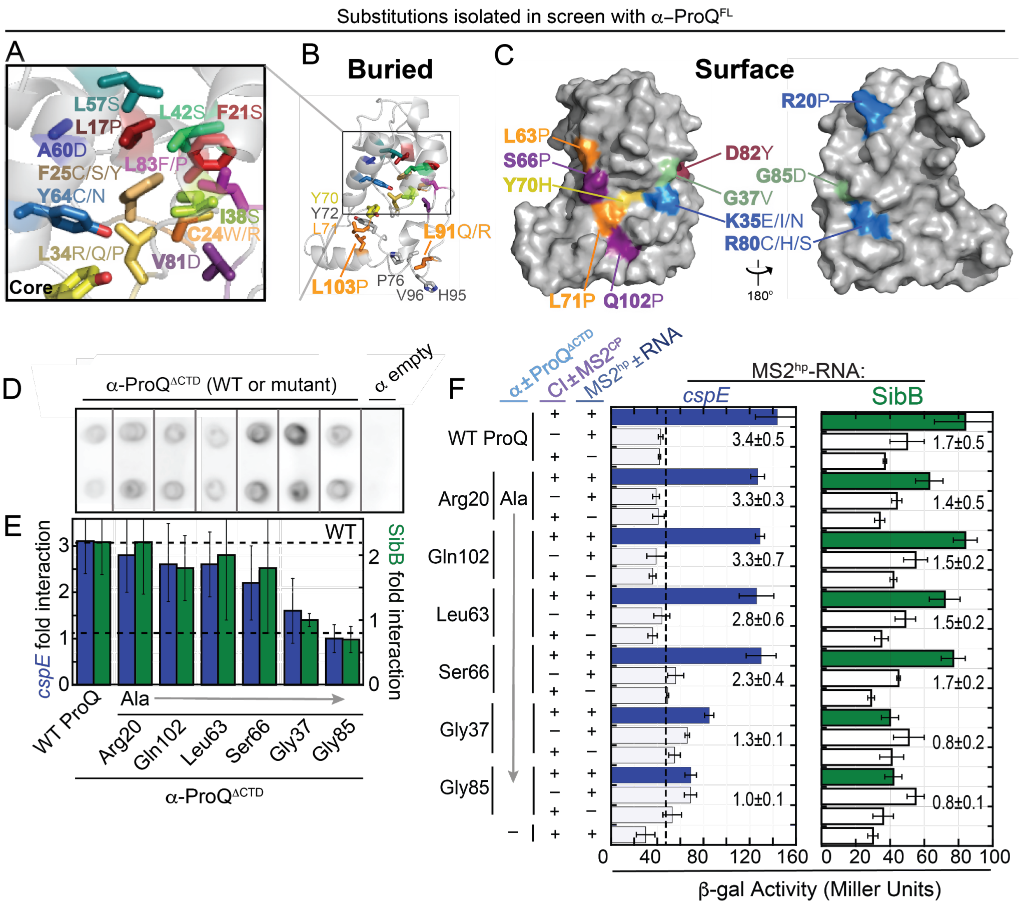

Supplementary Figure 10. Results and validation of unbiased genetic screen. (A) Surface and (B) cartoon representation of ProQ NTD structure (PDB ID: 5nb9),(9) viewed from concave face, showing buried residues at which substitutions were found to disrupt RNA binding in B3H screen with mutagenized α-ProQ^{FL} plasmids (Table 1). Inset shows core residues identified in the RNA-binding screen. Each residue is shown in stick representation, and the substitution(s) identified in the screen is/are indicated with labels of matching color. (B) Additional buried residues at interface of H5 helix and core are shown as orange sticks and labeled with the substitution identified in the screen. Neighboring hydrophobic side chains not identified in the screen are shown as grey sticks. (C) Surface representation of ProQ NTD structure, viewed from concave face (left) and convex face (right), with positions of surface-exposed residues at which substitutions were found to disrupt RNA binding in B3H screen with mutagenized α-ProQ^{FL} plasmids. Residue coloring: basic, blue; hydrophobic, orange; aromatic, yellow; acidic, red; polar: purple; glycine: green. Results of (D) dot-blot assay and (E) β-galactosidase (β-gal) assays testing the effects of alanine substitutions at residues implicated in screen. β-gal assays were performed with *Δhfq* reporter strain cells containing three compatible plasmids: one that encoded λCI or the CI-MS2^{CP} fusion protein, another that encoded the α-ProQ^{ΔCTD} fusion protein (wild-type, WT, or the indicated mutant), and a third that encoded a hybrid RNA (MS2^{hp}-*cspE* or MS2^{hp}-SibB) or an RNA that contained only the MS2^{hp} moiety. The cells were grown in the presence of 0.2% arabinose and 50 μM IPTG. Lysates were spotted on nitrocellulose and blotted with anti-ProQ antibody, and also interrogated for β-gal activity (see Methods). The bar graph shows the fold-stimulation over basal levels as averages and standard deviations of values collected from three independent experiments conducted in triplicate across multiple days. (F) Absolute β-gal values of a representative dataset from which fold-stimulation over basal levels were calculated for data presented (E). Bar graphs show absolute β-gal values as the averages and standard deviations of biological triplicate measurements. Each interaction is labeled with the fold-stimulation over basal levels (see Methods).

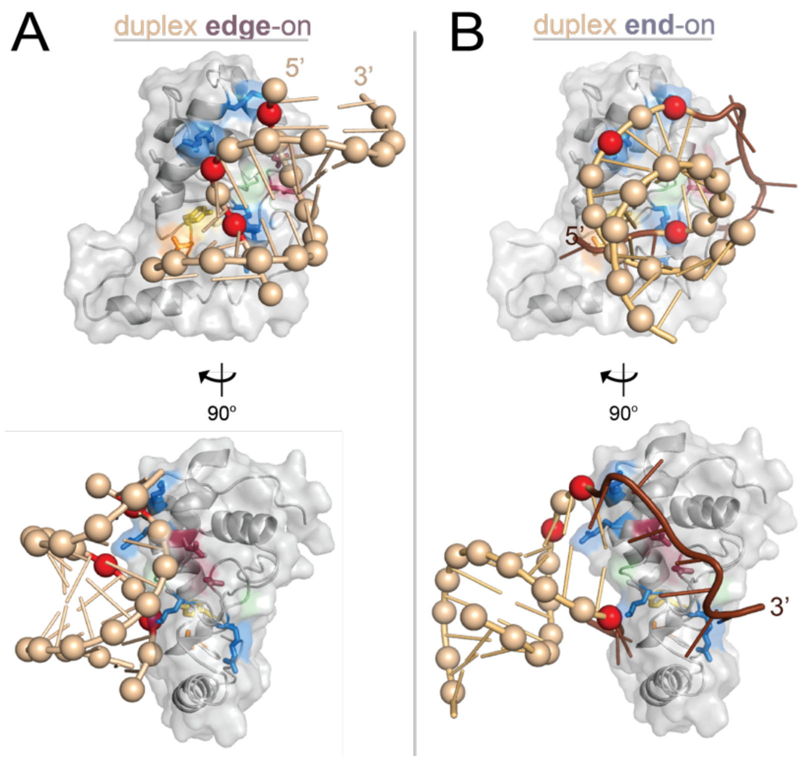

Supplementary Figure 11. Preliminary structural models for ProQ-NTD recognition of dsRNA and nearby ssRNA. The NTD/FinO-domain of ProQ is shown with an RNA duplex interacting with the concave face in an (A) edge-on or (B) end-on manner. (Models are presented as in Fig 5D from the concave face (top) and from a 90° rotation (bottom) from the β-3,4-hairpin-containing face of the protein. dsRNA is shown as a tan cartoon with phosphates as spheres; ssRNA at 5' and 3' base of hairpin (B) is colored brown. Three phosphates within the A-form duplex that have a suitable geometry to interact with basic residues (Lys35, Lys54, and Arg58) are colored in red. ProQ and residues are colored as in Fig 5 with side chains of RNA-binding residues shown as sticks. See Fig 5 legend for information about docking of RNA and protein to generate models.

A

Ec ProQ	G37	K35	D41	K54	R58	Y70	L71	R80	D82	G85
F' FinO	G99	ala	ala (-1: D102)	his (+1: K118)	R121	Y133	L134	R143	D145	G148
Nm NMB1681	G47	ala	D51	ala	ala (+1: R66)	Y77	L78	R87	D89	asn
Lp RocC	G52	K50	D56	K71	R75	Y87	L88	R97	D99	G102
*Lp*1663	G40	K37	D44	K59	R63	Y75	L76	R85	D87	G90

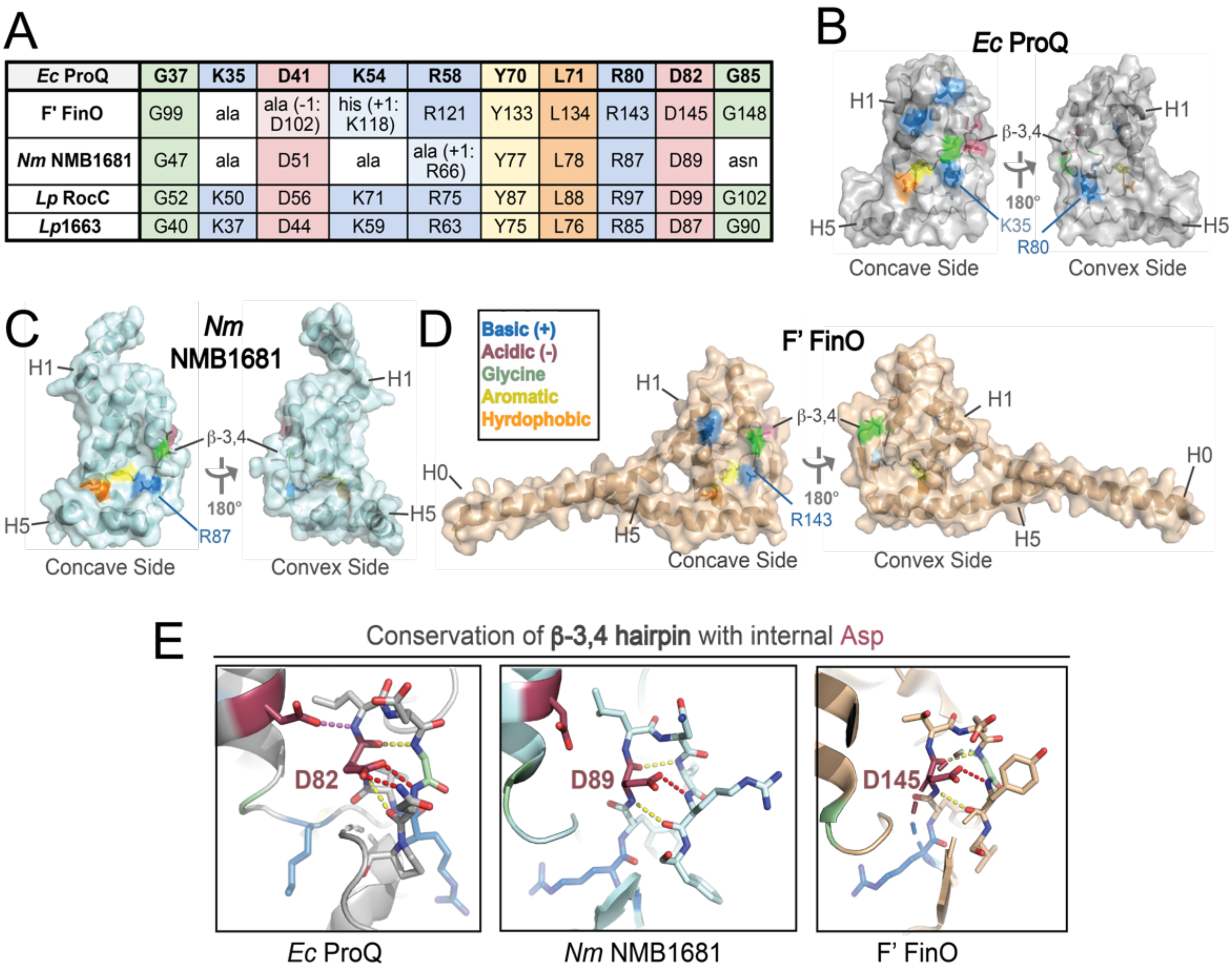

Supplementary Figure 12. Conservation of *E. coli* ProQ RNA-binding residues across other FinO-domain proteins. (A) The 10 residues identified in this work as important for RNA binding in *E. coli (Ec)* ProQ, whether from site-directed mutagenesis or a forward genetic screen, are listed in the top row, shaded by amino-acid character (basic, blue; hydrophobic, orange; aromatic, yellow; acidic, red; glycine: green). The corresponding residues in the *Ec* F' FinO protein, *N. meningitidis (Nm)* NMB1682, *L. pneumophila (Lp)* RocC and Lp1663 are listed in the rows below, based on a previous alignment. (10) If a residue is not conserved in the alignment, the amino acid in its place is listed. If a corresponding residue is present in the alignment at a position shifted by a single amino acid (-1/+1), that residue number is also indicated. Surface and cartoon representation of (B) *Ec* ProQ NTD NMR structure (PDB ID: 5nb9),(9) and crystal structures of (C) *Nm* NMB1682 (PDB ID: 3MW6),(5) or (D) *Ec* F' FinO protein (PDB ID: 1DVO),(3) viewed from concave face (left) and convex face (right), showing residues identified in this study as necessary for strong RNA interactions *in vivo,* or corresponding conserved residues, as listed in (A). Residues are shown in stick representation underneath a transparent surface, colored as above. Positively charged residues at corresponding positions to Arg80 are labeled. In both FinO and NM1681 crystal structures, this Arg residue has been modeled to be on the concave surface of the FinO-domain, in contrast to its position on the convex surface in the *Ec* ProQ NMR structure. In the latter structure, K35 occupies a similar position on the concave face as the arginine in the other structures.(3, 5) (E) Close-up representation of β-3,4 hairpin in stick representation, colored by element as above. Potential hydrogen bonds are shown as dashed lines, colored based on whether they involve only the polypeptide backbone (yellow), or Asp82 or analogous residue (red) or Asp41 (purple).

Supplementary Table S1. Plasmids used in this study. Listed in alphabetical order by plasmid name.

Name	Description	Details	Reference/ Source	Figures
pACλCI	empty vector	Encodes full-length λCI under the control of the *lacUV5* promoter; confers CamR	(1)	throughout
pBrα	empty vector	Encodes residues 1-248 of alpha fused under the control of tandem *lpp* and *lacUV5* promoters; confers AmpR	(1)	throughout
pCH1	pCDF-1XMS2^{hp}	pCDF-pBAD-MS2hp-XmaI-HindIII; remove a 1xMS2 RNA hairpin from pKB845; confers SpcR	This study (oCH1, oCH2; see Methods)	throughout
pCH6	pCDF-1XMS2^{hp}-ChiX	*E. coli chiX* cloned behind MS2hp in pCH1 between XmaI/HindIII sites; sRNA encodes its own terminator; confers SpcR	This study (oKB1190, oKB1991)	Fig 2
pCH9	pCDF-1XMS2^{hp}-OxyS	*E. coli oxyS* cloned behind MS2hp in pCH1 between XmaI/HindIII sites; sRNA encodes its own terminator; confers SpcR	This study (oKB1996, oKB1197)	Fig 2
pCH13	pCDF-1XMS2^{hp}-ArcZ	*E. coli arcZ* cloned behind MS2hp in pCH1 between XmaI/HindIII sites; sRNA encodes its own terminator; confers SpcR	This study (oKB1211, oKB1212)	Fig 2
pCW17	pAC-p$_{constitutive}$-λCIMS2^{CP}	Encodes residues 1-248 of CI fused via an AAAEFPGIHPGM linker to a multimerization-resistant MS2 coat protein; transcription of fusion protein driven by a constitutive promoter; confers CamR	This study (see Methods)	throughout
pFW11-tet OL2	pFW11tet-O$_L$2-62-lacZ	pFW11-derivative plasmid (2) bearing test promoter p$_{lac}$O$_L$2-62 fused to lacZ; confers TetR	Analogous to (4, 11)	N/A
pHL6	pCDF-1XMS2^{hp}-T$_{trpA}$	Intrinsic terminator from *trpA* operon cloned behind MS2hp in pCH1 between XmaI/HindIII sites; confers SpcR	This study (oHL19, oHL20; from pCH1)	Fig 2
pKB817	pBrα-Hfq	Encodes residues 1-248 of alpha fused via three alanine residues to full-length wild-type *E. coli* Hfq; confers AmpR	(11)	Fig 2C, Fig S2B
pKB822	pCDF-empty	CDF origin and spectinomycin resistance from pCDF1b combined with araC-pBAD-rrnB	(11)	Fig S2

		sequences from pBAD33; Confers SpcR		
pKB949	pBrα-ProQ^{FL}	Encodes residues 1-248 of alpha fused via three alanine residues to full-length wild-type *E. coli proQ*; confers AmpR	This study (oKB1225, oKB1226)	Fig 3, Table 1
pKB951	pBrα-ProQ$^{NTD+12aa}$	Encodes residues 1-248 of alpha fused via three alanine residues to residues 1-131 of wild-type *E. coli proQ*; TAA stop codon after *proQ* added through PCR; confers AmpR	This study (oKB1225, oKB1227)	Fig 3
pKB955	pBrα-ProQ$^{\Delta CTD}$	Encodes residues 1-248 of alpha fused via three alanine residues to residues 1-176 of wild-type *E. coli proQ*; TAA stop codon after *proQ* added through PCR; confers AmpR	This study (oKB1225, oKB1229)	throughout
pKB989	pAC-plac-λCIMS2^{CP}	Encodes residues 1-248 of CI fused via an AAAEFPGIHPGM linker to a multimerization-resistant MS2 coat protein; transcription of fusion protein driven by a lacUV5 promoter; confers CamR	(11)	N/A
pKB1067	pFW11tet-O$_L$2-83-lacZ	pFW11-derivative plasmid(2) bearing test promoter p$_{lac}$ *O$_L$2-83-lacZ* ; confers TetR pFW11$_{tet}$,	This study (see Methods; oKB1362, oKB1363, oKB1366, oKB1367)	N/A
pSP10	pCDF-1XMS2^{hp}-*cspE* 3'UTR	*E. coli* 3'UTR of *cspE* cloned behind MS2hp in pCH1 between XmaI/HindIII sites; mRNA encodes its own terminator; confers SpcR	This study (oSP10, oSP11)	throughout
pSP14	pCDF-1XMS2^{hp}-SibB	*E. coli sibB* cloned behind MS2^{hp} in pCH1 between XmaI/HindIII sites; sRNA encodes its own terminator; confers SpcR	This study (oSP16, oSP17)	throughout
pSP25	pCDF-1XMS2^{hp}-RyjB	*E. coli ryjB* (140 nts) cloned behind MS2hp in pCH1 between XmaI/HindIII sites; confers SpcR	This study (oSP21, oSP23)	Fig 2
pSP56	pCDF-1XMS2^{hp}-*fbaA* 3'UTR	*E. coli* 3'UTR (terminal 91 nt of transcript, beginning +1038 after ATG) of *fbaA* cloned behind MS2^{hp} in pCH1 between XmaI/HindIII sites; mRNA encodes its own terminator; confers SpcR	This study (oSP72, oSP76)	Fig 2
pSP87	pCDF-1XMS2^{hp}-MgrR	*E. coli mgrR* cloned behind MS2^{hp} between XmaI/HindIII sites; sRNA its own terminator; confers SpcR	This study (oKB1109, oKB1110)	Fig 2

pSP90	pBrα-ProQ^{NTD}	Encodes residues 1-248 of alpha fused via three alanine residues to residues 1-119 of wild-type *E. coli proQ*; TAA stop codon after *proQ* added through PCR; confers AmpR	This study (oKB1225, oSP126)	Fig 3
pSP92	pBrα-ProQ^{CTD}	Encodes residues 1-131 of alpha fused via three alanine residues to residues 181-232 of wild-type *E. coli proQ*; confers AmpR	This study (oSP128, oKB1226)	Fig 3
**Plasmids cloned for site-directed mutagenesis**				
pCG1	pBrα-ProQ$^{\Delta CTD}$-K35A	*proQ* K35A mutation introduced to pKB955; confers AmpR	This study	Fig 4D
pCG3	pBrα-ProQ$^{\Delta CTD}$-L71I	*proQ* L71I mutation introduced to pKB955; confers AmpR	This study (oSP90, oCG6)	Fig 4E
pOS18	pBrα-ProQ$^{\Delta CTD}$-D82N	*proQ* D82N mutation introduced to pKB955; confers AmpR	This study (oOS27, oSP80)	Fig 4F
pSP101	pBrα-ProQ$^{\Delta CTD}$-D41A	*proQ* D41A mutation introduced to pKB955; confers AmpR	This study (oSP77, oSP78)	Fig 4F
pSP102	pBrα-ProQ$^{\Delta CTD}$-D41E	*proQ* D41E mutation introduced to pKB955; confers AmpR	This study (oSP77, oSP78)	Fig 4F
pSP103	pBrα-ProQ$^{\Delta CTD}$-D82A	*proQ* D82A mutation introduced to pKB955; confers AmpR	This study (oSP79, oSP80)	Fig 4F
pSP104	pBrα-ProQ$^{\Delta CTD}$-D82E	*proQ* D82E mutation introduced to pKB955; confers AmpR	This study (oSP79, oSP80)	Fig 4F
pSP107	pBrα-ProQ$^{\Delta CTD}$-K54A	*proQ* K54A mutation introduced to pKB955; confers AmpR	This study (oSP83, oSP84)	Fig 4D
pSP111	pBrα-ProQ$^{\Delta CTD}$-Y70F	*proQ* Y70F mutation introduced to pKB955; confers AmpR	This study (oSP87, oSP88)	Fig 4E
pSP112	pBrα-ProQ$^{\Delta CTD}$-Y70S	*proQ* Y70S mutation introduced to pKB955; confers AmpR	This study (oSP87, oSP88)	Fig 4E
pSP113	pBrα-ProQ$^{\Delta CTD}$-L71V	*proQ* L71V mutation introduced to pKB955; confers AmpR	This study (oSP89, oSP90)	Fig 4E
pSP114	pBrα-ProQ$^{\Delta CTD}$-L71A	*proQ* L71A mutation introduced to pKB955; confers AmpR	This study (oSP90, oSP110)	Fig 4E
pSP117	pBrα-ProQ$^{\Delta CTD}$-R80A	*proQ* R80A mutation introduced to pKB955; confers AmpR	This study (oSP92, oSP111)	Fig 1E,F Fig 2B, Fig 4D
pSP136	pBrα-ProQ$^{\Delta CTD}$-K75A	*proQ* K75A mutation introduced to pKB955; confers AmpR	This study (oSP134, oSP135)	Fig 4D
pSP137	pBrα-ProQ$^{\Delta CTD}$-R58A	*proQ* R58A mutation introduced to pKB955; confers AmpR	This study (oSP86, oSP137)	Fig 4D
pSP125	pBrα-ProQ$^{\Delta CTD}$-G85A	*proQ* G85A mutation introduced to pKB955; confers AmpR	This study (oSP99, oSP100)	Fig S10D-F
pSP128	pBrα-ProQ$^{\Delta CTD}$-K107A	*proQ* K107A mutation introduced to pKB955; confers AmpR	This study (oSP103, oSP104)	Fig 4D
pSP139	pBrα-ProQ$^{\Delta CTD}$-Q102A	*proQ* Q102A mutation introduced to pKB955; confers AmpR	This study (oSP141, oSP142)	Fig S10D-F

pSP147	pBrα-ProQ$^{\Delta CTD}$-R20A	*proQ* R20A mutation introduced to pKB955; confers AmpR	This study (oSP159, oSP160)	Fig S10D-F
pSP148	pBrα-ProQ$^{\Delta CTD}$-S66A	*proQ* S66A mutation introduced to pKB955; confers AmpR	This study (oCG9, oCG10)	Fig S10D-F
pSP149	pBrα-ProQ$^{\Delta CTD}$-G37A	*proQ* G37A mutation introduced to pKB955; confers AmpR	This study (oSP163, oSP132)	Fig S10D-F
pSP153	pBrα-ProQ$^{\Delta CTD}$-L63A	*proQ* L63A mutation introduced to pKB955; confers AmpR	This study (oSP164, oSP165)	Fig S10D-F

Supplementary Table S2. Strains used in this study.

Name	Relevant Details	Antibiotic Resistanc e	Reference/ Source	Figures
NEB 5alpha F'Iq	*E. coli lacIq* host strain for plasmid construction	TetR	New England Biolabs	N/A
KB480	FW102 harboring an F' episome bearing tetracycline resistance and test promoter *placO$_L$2-62* fused to *lacZ*	TetR, StrR	This study (see Methods)	Fig 1B, Fig S2
KB483	FW102 *hfq::kan* harboring F' episome bearing tetracycline resistance and test promoter *placO$_L$2-62* fused to *lacZ*	TetR, KanR, StrR	This study (see Methods)	throughout
KB496	FW102 *hfq::kan*	KanR; StrR	(11)	N/A
KB511	FW102 harboring an F' episome bearing tetracycline resistance and test promoter *placO$_L$2-82* fused to *lacZ*;	TetR, StrR	This study (see Methods)	N/A
SP2	FW102 *proQ::kan* harboring an F' episome bearing tetracycline resistance and test promoter *placO$_L$2-62* fused to *lacZ*	TetR, KanR, StrR	This study (see Methods)	Fig 1C
SP5	FW102 *hfq::kan* harboring an F' episome bearing tetracycline resistance and test promoter *placO$_L$2-83* fused to *lacZ*;	TetR, KanR, StrR	This study (see Methods)	Genetic Screen (Table 1); results in Table S6 show verification in KB483.

Supplementary Table S3. Oligonucleotides used in this study. Listed in alphabetical order by name. M = C or A; Y = U or C; N = A/U/G/C

Name	Description	Used for	Sequence
oCG1	F ProQ D82A	Mutagenesis PCR: pSP103	ACGCGTGTCGCTCTTGACGGC
oCG2	R ProQ D82A	Mutagenesis PCR: pSP103	TGCGCCGGGTTTAACACC
oCG3	F ProQ K35A	Mutagenesis PCR: pCG1	GCGTCCGCTGGCAATCGGTATTTTTC

oCG4	R ProQ K35A	Mutagenesis PCR: pCG1	GCTTCACCTTCCGCACTG
oCG6	F ProQ L71I	Mutagenesis PCR: pCG3	CTGGCGTTATATTTACGGTGTTA
oCG7	F ProQ L91A	Mutagenesis PCR: pCG4	ATGCGGTGAGGCGGACGAGCAAC
oCG9	R ProQ L91A	Mutagenesis PCR: pCG4	GGGTTGCCGTCAAGATCG
oCG9	F ProQ S66A	Mutagenesis PCR: pSP148	TCTCTACACTGCGAGCTGGCG
oCG10	R ProQ S66A	Mutagenesis PCR: pSP148	CGTAAAGCGGATCGCAATTG
oCH1	F EcoRI	PCR: pCH1	AATTCAGAAAACATGAGGATCACCCATGTCTGCAGC
oCH2	R XmaI	PCR: pCH1	CCGGGCTGCAGACATGGGTGATCCTCATGTTTTCTG
oCW6	F pKB989 backbone	Gibson assembly: pCW17	GCTAGCGCATGCCACACAGGAAACAGCGTATGAGCAC
oCW7	R pKB989 backbone	Gibson assembly: pCW17	CTCGAGGCCTGGGGTGCCTAATGAGTGAG
oCW18	F constitutive promoter	Gibson assembly: pCW17 -10	ACCCCAGGCCTCGAGTTTACGGCTAGCTCAGTCCTAGGTATAGTGCTAGCGCATGCCACA
oCW19	R constitutive promoter	Gibson assembly: pCW17 -10	TGTGGCATGCGCTAGCACTATACCTAGGACTGAGCTAGCCGTAAACTCGAGGCCTGGGGT
oCW28	F pCW17 -35	Mutagenesis PCR: pCW17	CCAGGCCTCGAGACGATAGCTAGCTCAGTC
oCW29	R pCW17 -35	Mutagenesis PCR: pCW17	GACTGAGCTAGCTATCGTCTCGAGGCCTGG
oHL19	F T_{TrpA}	Mutagenesis PCR: pHL6	AGCGGGCTTTTTTTTCAGCTTGGCTGTTTTGGCGGATGAG
oHL20	R T_{trpA}	Mutagenesis PCR: pHL6	CATTAGGCGGGCTAAGCTTGCATGCCTGCAGGTCCCGGGC
oKB1190	F XmaI ChiX	PCR: pCH6	CGCCAATAGCGATATTGGCCATTTTTTTAAGCTTGGCGG
oKB1191	R HindIII ChiX	PCR: pCH6	CCGGCCAAGCTTAAAAAAATGGCCAATATCGCTATTGGC
oKB1196	F XmaI OxyS	PCR: pCH9	TCCCCCCGGGGAAACGGAGCGGCACCTC
oKB1197	R HindIII OxyS	PCR: pCH9	CCGGCCAAGCTTAAAAAAAAGCGGATCCTGGAGATCC
oKB1211	F XmaI ArcZ	PCR: pCH13	TCCCCCCGGGGTGCGGCCTGAAAAACAGTGC
oKB1212	R HindIII ArcZ	PCR: pCH13	CCGGCCAAGCTTAAAAAATGACCCCGGCTAGACC
oKB1109	F XmaI MgrR	PCR: pSP87	TCCCCCCGGGGATTCGTTATCAGTGCAGGAAAATGCC
oKB1110	R HindIII MgrR	PCR: pSP87	CCGGCCAAGCTTAAAAAAAACCGCCAGTAAACCGGC

oKB1225	F NotI *proQ*	PCR: pKB949, pKB955, pSP90, pKB951	ATAAGAATGCGGCCGCAGAAAATCAACCTAAGTTGAATAGCAGTAAAG
oKB1226	R BamHI *proQ*	PCR: pKB949, pSP92	CTTCGGATCCTCAGAACACCAGGTGTTCTGCGC
oKB1227	R BamHI *proQ* NTD+12aa	PCR: pKB951	CTTCGGATCCTTATTTCTCACCAGCAGTTGCG
oKB1229	R BamHI *proQ* ΔCTD	PCR: pKB955	CTTCGGATCCTTACTGTTCTTCGCGAGGTGCTTTTAC
oKB1362	F EcoRI	PCR: pKB1067	GGCCGGGAATTCTTCCACCGGCGG
oKB1363	R HindIII	PCR: pKB1067	CCGGCCAAGCTTGGCTGCAGGTCG
oKB1366	F overlap +21bp	PCR: pKB1067	CAACACCGCCAGAGATAGCTGCCACGGTGCCCGACCGTCCTGAGGCACCCCGGGC
oKB1367	R overlap +21bp	PCR: pKB1067	GCCCGGGGTGCCTCAGGACGGTCGGGCACCGTGGCAGCTATCTCTGGCGGTGTTG
oOS27	F ProQ D82N	PCR: pOS18	AACGCGTGTCAATCTTGACGGC
oSP10	F XmaI *cspE* 3'UTR	PCR: pSP93, pSP10	GGCCGGCCCGGGCAAAGGCCCTTCTGCTGCAAAC
oSP11	R HindIII *cspE* 3'UTR	PCR: pSP93, pSP10	CCGGCCAAGCTTAAAAAAAACCCGCTGATTAAGCGGGT
oSP16	F XmaI SibB	PCR: pSP14	GGCCGGCCCGGGGAGGGTAGAGCGGGGTTTCC
oSP17	R HindIII SibB	PCR: pSP14	CCGGCCAAGCTTGGAAAGCCCCTCCCGAGG
oSP21	F XmaI RyjB	PCR: pSP25	GGCCGGCCCGGGTCATCCGTCGTTGACTCCATGCC
oSP23	R HindIII RyjB	PCR: pSP25	CCGGCCAAGCTTCGATAAGATGGGGATAAGCAGAGCGCTTAT
oSP76	F XmaI *fbaA* 3'UTR	PCR: pSP56	GGCCGGCCCGGGGGAGAAAGCATTCCAGGAACTGAACG
oSP72	R HindIII *fbaA* 3'UTR	PCR: pSP56	CCGGCCAAGCTTAAAAAAAGACCCGCAGAGCGGG
oSP77	F ProQ D41 (A/E)	Mutagenesis PCR: pSP101, pSP102	ATTTTTCAGGMGTTGGTCGATCGTGTTGC
oSP78	R ProQ D41 (A/E)	Mutagenesis PCR: pSP101, pSP102	ACCGATTTTCAGCGGACG
oSP79	F ProQ D82 (A/E)	Mutagenesis PCR: pSP104	AACGCGTGTCGMGCTTGACGGCAAC
oSP80	R ProQ D82 (A/E/N)	Mutagenesis PCR: pSP104	GCGCCGGGTTTAACACCG
oSP83	F ProQ K54 (A/E)	Mutagenesis PCR: pSP107	GAACCTGAGCGMGACGCAATTGCG
oSP84	R ProQ K54 (A/E)	Mutagenesis PCR: pSP107, pSP145	ATTTCCCCAGCAACACGA
oSP86	R ProQ R58A	Mutagenesis PCR: pSP137, pSP150	TTGCTCAGGTTCATTTCC
oSP87	F ProQ Y70 (F/S)	Mutagenesis PCR: pSP111, pSP112	GAGCTGGCGTTYTCTTTACGGTG

oSP88	R ProQ Y70 (F/S)	Mutagenesis PCR: pSP111, pSP112	GAAGTGTAGAGACGTAAAGC
oSP89	F ProQ L71 (A/V)	Mutagenesis PCR: pSP113,	CTGGCGTTATGNTTACGGTGTTAAAC
oSP90	R ProQ L71 (A/V)	Mutagenesis PCR: pSP113, pSP114, pCG3	CTCGAAGTGTAGAGACGTAAAG
oSP92	R ProQ R80A	Mutagenesis PCR: pSP117	GGTTTAACACCGTAAAGATAAC
oSP99	F ProQ G85 (D/A)	Mutagenesis PCR: pSP125	CGATCTTGACGMTAACCCATGCGGTGAGCTGGA C
oSP100	R ProQ G85 (D/A)	Mutagenesis PCR: pSP125	ACACGCGTTGCGCCGGGT
oSP103	F ProQ K107 (A/E)	Mutagenesis PCR: pSP128	TGAAGAAGCGGMGGCGCGTGTTCAGG
oSP104	R ProQ K107 (A/E)	Mutagenesis PCR: pSP128	AGCTGCTTGCGAGCATGC
oSP110	F ProQ L71A	Mutagenesis PCR: pSP114	CTGGCGTTATGCTTACGGTGTTAAAC
oSP111	F ProQ R80A	Mutagenesis PCR: pSP117, pSP151, pSP152, pSP160	CGGCGCAACGGCGGTCGATCTTG
oSP126	R BamHI proQ NTD	PCR: pSP90	CCGGCCGGATCCTTACGCTTGCTGTTCAGCACG
oSP128	F NotI I *proQ* CTD	PCR: pSP92	GAATGCGGCCGCATCTGACATTTCAGCTCTGAC TGTCGGA
oSP135	F ProQ K75A	Mutagenesis PCR: pSP136	TTACGGTGTTGCACCCGGCGCAA
oSP134	R ProQ K75A	Mutagenesis PCR: pSP136	AGATAACGCCAGCTCGAAG
oSP137	F ProQ R58A	Mutagenesis PCR: pSP137	AACGCAATTGGCATCCGCTTTAC
oSP141	F ProQ Q102A	Mutagenesis PCR: pSP137	TGCTCGCAAGGCGCTTGAAGAAG
oSP142	R ProQ Q102A	Mutagenesis PCR: pSP137	TGCTCTACATGTTGCTCG
oSP163	F ProQ G37A	Mutagenesis PCR: pSP149	GCTGAAAATCGCTATTTTTCAGGATTTGGTCGAT CG
oSP132	R ProQ G37A	Mutagenesis PCR: pSP149	GGACGCGCTTCACCTTCC
oSP159	F ProQ R20A	Mutagenesis PCR: pSP147	TCTGGCCGAAGCTTTTCCCCACTG
oSP160	R ProQ R20A	Mutagenesis PCR: pSP147	AACGCGATTACTTCTTTAC
oSP164	F ProQ L63A	Mutagenesis PCR: pSP153	CGCTTTACGTGCCTACACTTCGAG
oSP165	R ProQ L63A	Mutagenesis PCR: pSP153	GATCGCAATTGCGTTTTG

Supplementary Table S4. Sequences of Hybrid RNAs used in B3H assay. Light blue letters indicate linker and cloning sites. Dark blue letters indicate the 21-nt MS2^{hp} sequence. Black letters indicate the bait RNA sequence inserted between XmaI and HindIII sites.

Plasmid	Description	Sequence
pSP10	pCDF-1XMS2^{hp}-*cspE* 3'UTR	AGAAAACAUGAGGAUCACCCAUGUCUGCAGCCCGGG<br>CAAAGGCCCUUCUGCUGCAAACGUAAUCGCUCUGUA<br>AGAUACGUCAGCAAGAAUUCAAAACCCGCUUAAUCAG<br>CGGGUUUUUUUU
pSP14	pCDF-1XMS2^{hp}-SibB	AGAAAACAUGAGGAUCACCCAUGUCUGCAGCCCGGG<br>GAGGGUAGAGCGGGGUUUCCCCCGCCCUGGUAGUC<br>UUAGUAAGCGGGGAAGCUUAUGACUAAGAGCACCAC<br>GAUGAUGAGUAGCUUCAUCAUGACCCUUUCCUUAUU<br>UAUGGCCCCUUCCUCGGGAGGGGCUUUCC
pSP25	pCDF-1XMS2^{hp}-RyjB	AGAAAACAUGAGGAUCACCCAUGUCUGCAGCCCGGG<br>UCAUCCGUCGUUGACUCCAUGCCGAUUCGGGUUAAU<br>CUGGUAGCGAUCCCCGUCGAUACUUUUGACGAAGGC<br>GGCAGGGAUCGCAGAAGGGUUAUUGCUCUUUGCCGG<br>GGAUAAGCGCUCUGCUUAUCCCCAUCUUAUCG
pSP56	pCDF-1XMS2^{hp}-*fbaA* 3'UTR	AGAAAACAUGAGGAUCACCCAUGUCUGCAGCCCGGG<br>GGAGAAAGCAUUCCAGGAACUGAACGCGAUCGACGU<br>UCUGUAAGAUAUUCCUUUCUGCUUAUCUCAAGGCCC<br>GCUCUGCGGGUCUUUUUUU
pSP87	pCDF-1XMS2^{hp}-MgrR	AGAAAACAUGAGGAUCACCCAUGUCUGCAGCCCGGG<br>GAUUCGUUAUCAGUGCAGGAAAAUGCCUGUUAGCGU<br>AAAAGCAAAACACAAAUCUAUCCAUGCAAGCAUUCAC<br>CGCCGGUUUACUGGCGGUUUUUUUU
pCH6	pCDF-1XMS2^{hp}-ChiX	AGAAAACAUGAGGAUCACCCAUGUCUGCAGCCCGGG<br>ACACCGUCGCUUAAAGUGACGGCAUAAUAAUAAAAAA<br>AUGAAAUUCCUCUUUGACGGGCCAAUAGCGAUAUUG<br>GCCAUUUUUUU
pCH9	pCDF-1XMS2^{hp}-OxyS	AGAAAACAUGAGGAUCACCCAUGUCUGCAGCCCGGG<br>GAAACGGAGCGGCACCUCUUUUAACCCUUGAAGUCA<br>CUGCCCGUUUCGAGAGUUUCUCAACUCGAAUAACUA<br>AAGCCAACGUGAACUUUUGCGGAUCUCCAGGAUCCG<br>CUUUUUUUU
pCH13	pCDF-1XMS2^{hp}-ArcZ	AGAAAACAUGAGGAUCACCCAUGUCUGCAGCCCGGG<br>GUGCGGCCUGAAAAACAGUGCUGUGCCCUUGUAACU<br>CAUCAUAAUAAUUUACGGCGCAGCCAAGAUUUCCCUG<br>GUGUUGGCGCAGUAUUCGCGCACCCCGGUCUAGCCG<br>GGGUCAUUUUUU
pHL6	pCDF-1XMS2^{hp}-T_{trpA}	AGAAAACAUGAGGAUCACCCAUGUCUGCAGCCCGGG<br>ACCTGCAGGCATGCAAGCTTAGCCCGCCTAATGAGCG<br>GGCTTTTTTTT

Supplementary Table S5. Validation of dot-blot assay as an effective counter-screen to identify pBRα-*proQ* plasmids encoding α-ProQ^{FL} fusion proteins with low expression levels. B3H reporter-strain cells (SP5) containing the CI-MS2^{CP} adapter protein and either the MS2^{hp}-*cspE* hybrid RNA were transformed with an pBrα-*proQ*^{FL} mutagenesis library and plated appropriate on indicator medium to enable a clear distinction between blue positive-control colonies (containing the WT fusion proteins and the *cspE* hybrid RNA) and white negative-control colonies (instead containing a plasmid encoding α-empty). Ten white or pale colonies were picked, grown in liquid culture and ProQ-expression levels were determined by dot-blot assay and densitometry (see Methods and Fig S7). Plasmids were mini-prepped from 5 colonies with ProQ levels similar to those with an α-empty plasmid and 5 colonies with ProQ levels similar to WT α-ProQ controls. The *proQ* sequence from pBrα-*proQ*^{FL} plasmids in each colony was sequenced and compared to WT *proQ*. The consequences of each mutation present are indicated in terms of whether they lead to a premature stop codon and the location of each amino-acid substitution, including whether a mutation alters a codon to an in-frame stop codon (X) or introduces a frameshift (fs) that leads to stop codon (fsX) shortly thereafter.

ProQ expression levels	Mutant Name	Premature Stop Codon?	Consequence of Mutation
Low Levels (~α-empty)	0Mn-48	Yes	L42fsX
	0Mn-57	Yes	L61X
	0Mn-62	Yes	Q40fsX
	0Mn-67	Yes	N3fsX
	0Mn-89	Yes	Q40fsX
High Levels (~WT α-ProQ^{FL})	0Mn-52	No	Y70H
	0Mn-53	No	S66P
	0Mn-68	No	F25S
	0Mn-100	No	I38T; V43D
	0Mn-101	No	A15T; D82Y

Supplementary Table S6. Validation and Quantification of Results from Unbiased Screen. Results of β-galactosidase (β-gal) and dot-blot assays to confirm behavior of pBrα-*proQ*^{FL} plasmids isolated in forward genetic screen in KB483 reporter cells (carrying *O*$_L$*2-62-lacZ* reporter). Reporter-strain KB483 cells containing KB483 containing plasmids encoding *(i)* the CI-MS2^{CP} fusion protein, and *(ii)* either a MS2^{hp}-*cspE (cspE)* or MS2^{hp}-SibB (SibB) hybrid RNA were transformed with plasmids isolated from white/pale primary-screen colonies that passed the dot-blot counter-screen (see Methods) in order to introduce the mutant pBRα-*proQ*^{FL} plasmid contained in each colony. Cells were grown in triplicate in the presence of 0.2% arabinose and either 0 μM or 50 μM IPTG. Lysates from colonies containing MS2^{hp}-*cspE* were then assessed via dot-blot assay with an anti-ProQ antibody and densitometry was conducted to quantify the intensity of each dot. β-gal values from triplicate colonies were averaged and then normalized to the value from comparable colonies containing pBRα-empty as a negative control. This fold-stimulation value from each *proQ* mutant, along with average dot-blot intensity was then normalized to the values arising from WT α-ProQ (set to 1.0) and from α-empty (set to 0.0) in cells grown at 0 μM IPTG. The three values that were set to 1.0 are indicated in bold text within the table. Thus, the normalized expression levels (dot-blot intensity) and fold-stimulation of each mutant can be directly compared between IPTG concentrations. A small number of α–ProQ variants show lower expression levels than WT when both are induced with 0 μM IPTG, but induction of each variant with 50 μM IPTG leads to higher expression levels than WT α–ProQ at 0 μM IPTG. For each of these α–ProQ variants, induction with 50 μM IPTG, nevertheless results in lower B3H interaction for the variant than WT α–ProQ in the absence of IPTG. Errors reported for both sets of measurements represent one standard deviation from each mean value, propagated through each normalization step. For a description of variables listed under "Mutant Information," see Table 1.

**Mutant Information**					**Normalized β-gal Fold-Stimulation**				**Normalized Dot-Blot Intensity**	
**Resi**	**Loc**	**pBRα-*proQ*^{FL} Mutant Name**	**RNA**	**Variant**	**0 μM IPTG**		**50 μM IPTG**		**0 μM IPTG**	**50 μM IPTG**
					cspE	**SibB**	***cspE***	**SibB**		
				WT	**1.0±0.2**	**1.0±0.2**	0.9±0.2	0.9±0.4	**1.0±0.4**	2.7±1.2
				α empty	0.0	0.0	0.0	0.0	**0.0**	0.0
L17	core	0-Mn-56; 0.1Mn-SibB-55	both	L17P	0.2±0.1	0.2±0.2	0.1±0.1	0.3±0.1	0.7±0.4	1.4±0.5
R20	surface	0.1Mn-SibB-49	SibB	R20P	0.2±0.1	0.2±0.2	0.0±0.1	0.2±0.1	0.8±0.5	1.0±0.5
F21	core	0Mn-SibB-27; 0.1Mn-SibB-5	SibB	F21S	0.3±0.1	0.1±0.4	0.1±0.1	0.3±0.1	1.7±0.6	3.0±1.2
C24	core	0-Mn-55	*cspE*	C24W	0.2±0.1	0.2±0.2	0.1±0.1	0.3±0.1	0.7±0.3	2.7±1.9
		0.1Mn-SibB-16	SibB	C24R	0.2±0.1	0.2±0.2	0.1±0.1	0.5±0.2	1.0±0.4	3.4±1.2
F25	core	0.1Mn-50	*cspE*	F25C	0.2±0.1	0.2±0.2	0.1±0.1	0.4±0.2	0.9±0.6	2.0±1.5

		0-Mn-68; 0.1Mn-60; 0Mn-SibB-24	both	F25S	0.3±0.1	0.2±0.2	0.2±0.1	0.3±0.1	2.1±0.8	3.7±2.9
		0.1Mn-SibB-82	SibB	F25Y	0.4±0.1	0.3±0.2	0.3±0.1	0.3±0.1	1.3±0.5	2.6±2.4
L34	core	0-Mn-44	*cspE*	L34R	0.2±0.1	0.2±0.2	0.2±0.1	0.3±0.2	1.3±0.7	4.5±1.7
		0.1Mn-SibB-34	SibB	L34Q	0.2±0.1	0.2±0.2	0.1±0.1	0.3±0.1	0.7±0.5	2.1±1.5
		0.1Mn-SibB-50	SibB	L34P	0.3±0.1	0.2±0.2	0.1±0.1	0.2±0.1	1.7±0.9	3.6±2.2
K35	surface	0Mn-SibB-23	SibB	K35E	0.2±0.1	0.3±0.2	0.1±0.1	0.3±0.1	1.2±0.7	1.4±1.4
		0Mn-SibB-5	SibB	K35N	0.3±0.1	0.2±0.2	0.2±0.1	0.3±0.1	0.8±0.3	3.0±1.4
		0.1Mn-SibB-66	SibB	K35I	0.3±0.1	0.2±0.2	0.1±0.1	0.3±0.1	1.3±0.8	3.1±1.7
G37	surface	0Mn-SibB-17	SibB	G37V	0.3±0.1	0.1±0.2	0.1±0.1	0.2±0.1	2.0±1.0	2.8±1.1
I38	core	0-Mn-58	*cspE*	I38S	0.3±0.1	0.2±0.2	0.0±0.1	0.3±0.1	1.3±0.9	1.5±0.7
L42	core	0Mn-SibB-75	SibB	L42S	0.3±0.1	0.2±0.2	0.1±0.1	0.3±0.1	1.1±0.6	3.5±1.3
L57	core	0-Mn-14	*cspE*	L57S	0.3±0.1	0.2±0.2	0.1±0.1	0.2±0.1	1.1±0.4	3.5±1.7
A60	core	0-Mn-122	*cspE*	A60D	0.2±0.1	0.1±0.2	0.1±0.1	0.2±0.1	1.3±0.5	2.4±1.3
L63	surface	0.1Mn-SibB-60	SibB	L63P	0.3±0.1	0.3±0.2	0.1±0.1	0.1±0.1	1.0±0.5	1.5±0.6
Y64	core	0-Mn-99	*cspE*	Y64C	0.2±0.1	0.2±0.2	0.1±0.1	0.2±0.1	1.5±0.5	2.7±0.9
		0-Mn-136; 0Mn-SibB-61	both	Y64N	0.2±0.1	0.3±0.2	0.1±0.1	0.1±0.1	1.0±0.5	0.9±0.4
S66	surface	0.1Mn-SibB-44; 0-Mn-53	both	S66P	0.2±0.1	0.2±0.2	0.1±0.1	0.1±0.1	1.0±0.3	1.9±0.6
Y70	surface	0-Mn-52; 0.1Mn-61; 0Mn-SibB-8	both	Y70H	0.3±0.1	0.2±0.2	0.1±0.1	0.1±0.1	1.8±1.1	2.9±2.1
L71	surface	0-Mn-71; 0.1Mn-80; 0Mn-SibB-40	both	L71P	0.3±0.1	0.2±0.2	0.1±0.1	0.1±0.1	1.8±1.0	1.5±0.6
R80	surface	0-Mn-39	*cspE*	R80C	0.2±0.1	0.3±0.2	0.1±0.1	0.3±0.1	2.4±0.9	2.8±1.2
		0-Mn-98	*cspE*	R80H	0.4±0.1	0.3±0.2	0.1±0.1	0.2±0.1	2.9±1.3	4.4±1.7
		0.1Mn-SibB-43	SibB	R80S	0.2±0.1	0.2±0.2	0.1±0.1	0.2±0.1	1.0±0.4	1.7±0.8
V81	core	0-Mn-64; 0.1Mn-97; 0.1Mn-SibB-23	both	V81D	0.2±0.1	0.3±0.2	0.1±0.1	0.3±0.1	1.4±0.8	2.3±1.0
D82	surface	0.1Mn-151	*cspE*	D82Y	0.2±0.1	0.3±0.2	0.2±0.1	0.3±0.1	2.5±0.9	3.0±1.0
L83	core	0-Mn-158	*cspE*	L83F	0.2±0.1	0.3±0.2	0.3±0.1	0.2±0.1	2.2±1.6	2.0±1.2
		0Mn-SibB-57	SibB	L83P	0.3±0.1	0.2±0.2	0.3±0.1	0.2±0.1	1.3±0.8	1.9±1.0
G85	surface	0.1Mn-164; 0.1Mn-SibB-20	both	G85D	0.2±0.1	0.2±0.2	0.3±0.1	0.2±0.1	2.2±0.9	2.5±1.2
L91	buried	0Mn-SibB-32	SibB	L91Q	0.2±0.1	0.3±0.2	0.2±0.1	0.2±0.1	4.2±1.6	3.3±1.1
		0-Mn-113	*cspE*	L91R	0.1±0.1	0.3±0.2	0.2±0.1	0.3±0.1	2.9±1.2	2.2±0.7
Q102	surface	0Mn-SibB-78	SibB	Q102P	0.1±0.1	0.3±0.2	0.1±0.1	0.3±0.1	0.7±0.5	1.2±0.8
L103	buried	0.1Mn-SibB-32	SibB	L103P	0.2±0.1	0.3±0.2	0.3±0.1	0.3±0.1	3.5±1.2	2.8±1.1

SUPPLEMENTARY REFERENCES

1. Dove,S.L., Joung,J.K. and Hochschild,A. (1997) Activation of prokaryotic transcription through arbitrary protein-protein contacts. *Nature*, **386**, 627–630.

2. Whipple,F.W. (1998) Genetic analysis of prokaryotic and eukaryotic DNA-binding proteins in *Escherichia coli*. *Nucleic Acids Res*, **26**, 3700–3706.

3. Ghetu,A.F., Gubbins,M.J., Frost,L.S. and Glover,J.N. (2000) Crystal structure of the bacterial conjugation repressor finO. *Nat Struct Biol*, **7**, 565–569.

4. Deaconescu,A.M., Chambers,A.L., Smith,A.J., Nickels,B.E., Hochschild,A., Savery,N.J. and Darst,S.A. (2006) Structural basis for bacterial transcription-coupled DNA repair. *Cell*, **124**, 507–520.

5. Chaulk,S., Lu,J., Tan,K., Arthur,D.C., Edwards,R.A., Frost,L.S., Joachimiak,A. and Glover,J.N.M. (2010) N. meningitidis 1681 is a member of the FinO family of RNA chaperones. *RNA Biol*, **7**, 812–819.

6. Lorenz,R., Bernhart,S.H., Höner Zu Siederdissen,C., Tafer,H., Flamm,C., Stadler,P.F. and Hofacker,I.L. (2011) ViennaRNA Package 2.0. *Algorithms Mol Biol*, **6**, 26–14.

7. Kerpedjiev,P., Hammer,S. and Hofacker,I.L. (2015) Forna (force-directed RNA): Simple and effective online RNA secondary structure diagrams. *Bioinformatics*, **31**, 3377–3379.

8. Ashkenazy,H., Abadi,S., Martz,E., Chay,O., Mayrose,I., Pupko,T. and Ben-Tal,N. (2016) ConSurf 2016: an improved methodology to estimate and visualize evolutionary conservation in macromolecules. *Nucleic Acids Res*, **44**, W344–50.

9. Gonzalez,G.M., Hardwick,S.W., Maslen,S.L., Skehel,J.M., Holmqvist,E., Vogel,J., Bateman,A., Luisi,B.F. and Broadhurst,R.W. (2017) Structure of the *Escherichia coli* ProQ RNA-binding protein. *RNA*, **23**, 696–711.

10. Olejniczak,M. and Storz,G. (2017) ProQ/FinO-domain proteins: another ubiquitous family of RNA matchmakers? *Mol Microbiol*, **104**, 905–915.

11. Berry,K.E. and Hochschild,A. (2018) A bacterial three-hybrid assay detects *Escherichia coli* Hfq-sRNA interactions *in vivo*. *Nucleic Acids Res*, **46**, e12.
